# Comparative Analysis of Four Commercially Available Artificial Intelligence Software for Brain Volumetry

**DOI:** 10.3390/diagnostics16152383

**Published:** 2026-07-29

**Authors:** Gaspare Saltarelli, Giovanni Di Cerbo, Antonio Innocenzi, Marco De Chellis, Federico Bruno, Alessandra Splendiani, Ernesto Di Cesare

**Affiliations:** Department of Biotechnological and Applied Clinical Sciences, University of L’Aquila, 67100 L’Aquila, Italy; giovannidicerbo96@gmail.com (G.D.C.); antonio.innocenzi@yahoo.it (A.I.); dechellismarco94@gmail.com (M.D.C.); federico.bruno@univaq.it (F.B.); alessandra.splendiani@univaq.it (A.S.); ernesto.dicesare@univaq.it (E.D.C.)

**Keywords:** movement disorders, magnetic resonance imaging, brain volumetry, automated segmentation, artificial intelligence, image processing

## Abstract

**Background/Objectives:** Automated brain volumetry is increasingly integrated into the work-up of several neurological disorders, yet between-software variability may limit the interchangeability of absolute measures. This study aimed to assess inter-software variability, agreement, and interchangeability among four clinically available AI-based brain volumetry tools. **Methods:** We conducted a single-center, retrospective comparison of four clinically available tools (Neurophet AQUA^®^, icobrain dm^®^, mdbrain^®^, Pixyl^®^) applied to identical 3D T1-weighted MRI examinations (IR-FSPGR, 1 mm isotropic) acquired at 3 T in 47 adults referred for tremor evaluation. Absolute volumes (mL) were extracted for hippocampus, temporal, parietal, and frontal lobes, lateral ventricles, and total brain volume; the occipital lobe was analyzed where reported (three tools). Region-wise complete-case analyses were performed. Repeated-measures ANOVA with Bonferroni post hoc tests assessed systematic differences, and intraclass correlation coefficients (two-way mixed, consistency) were reported as single- and average-measure with 95% CIs to quantify agreement. **Results:** Across regions, all tests were significant (*p* < 0.001), indicating method-dependent scale offsets typically on the order of ~20–40%. A consistent pattern emerged: icobrain dm^®^ yielded the largest absolute volumes, Neurophet AQUA^®^ the lowest, mdbrain^®^ tended to be lower for parietal measurements, and Pixyl^®^ was generally intermediate. Agreement was structure-dependent: excellent for lateral ventricles (single-measure ICC ≈ 0.92 [0.85–0.96]; average-measure ICC ≈ 0.98 [0.96–0.99]); good-to-excellent for temporal and parietal lobes (average-measure ICC ≈ 0.83–0.84); moderate-to-good for hippocampus and total brain volume (TBV) (average-measure ICC ≈ 0.79–0.81), with only moderate single-measure agreement for TBV (ICC ≈ 0.51); and lowest for the frontal lobe (single-measure ICC ≈ 0.43 [0.23–0.65]; average-measure ICC ≈ 0.75 [0.54–0.88]). In the occipital lobe (three tools), Neurophet AQUA^®^ yielded substantially smaller values than icobrain dm^®^ and mdbrain^®^ (*p* < 0.0001). **Conclusions:** Overall, absolute volumetric outputs are not directly interchangeable across platforms, but relative subject ranking is largely preserved; platform-consistent analysis is advisable for longitudinal monitoring, and cross-site studies should implement calibration/harmonization before pooling data.

## 1. Introduction

Artificial intelligence (AI) has shown promising results in medicine, particularly in diagnostic neuroradiology. AI techniques—computer systems capable of performing tasks typically associated with human intelligence—are increasingly applied across neurological disorders, including movement disorders. Tremor syndromes, particularly essential tremor, encompass a heterogeneous clinical spectrum with motor and non-motor features [1]. Tremor is defined as an involuntary, rhythmic, oscillatory movement of a body part and is classified according to clinical characteristics and etiology [2]. Essential tremor is among the most common adult movement disorders, and a relevant proportion of patients may require escalation beyond standard symptomatic treatment when tremor remains functionally disabling [3,4]. From a pathophysiological perspective, essential tremor and Parkinsonian tremor may show overlapping clinical presentations while involving partially distinct motor networks, with converging evidence implicating cerebello-thalamo-cortical circuitry and cerebellar/subcortical abnormalities [5,6,7,8,9].

In patients with tremor, brain MRI is commonly used to exclude structural mimics, support differential assessment, and provide high-resolution anatomical data for quantitative analysis and, in selected medication-refractory cases, image-guided interventions such as magnetic resonance-guided focused ultrasound thalamotomy [10,11,12,13]. Automated brain volumetry can objectively quantify cortical, subcortical, and ventricular volumes from 3D T1-weighted MRI and may complement visual assessment by providing reproducible regional metrics.

In recent years, multiple AI-driven software tools for brain volumetry have been developed and are increasingly available for clinical use and incorporation into routine practice [14,15,16]. These tools—including the four packages used in the present study (mdbrain^®^, icobrain dm^®^, Neurophet AQUA^®^, and Pixyl^®^)—automatically quantify gray and white matter volumes, assess regional brain volumes, and report quantitative metrics complemented by normative reference values (e.g., age-adjusted percentiles from control populations) [15,16,17]. More broadly, automated neuroimaging pipelines rely on segmentation and parcellation procedures that can generate reproducible regional metrics but may differ according to atlas definitions and algorithmic implementation [18]. Accordingly, their use enables a more objective evaluation of patient-level brain structure, with potential benefits for longitudinal monitoring and for studies requiring harmonized quantitative MRI outputs.

A useful parallel can be drawn with automated perfusion post-processing software in acute ischemic stroke. In that setting, several commercial platforms are routinely used to estimate ischemic core, hypoperfused tissue, penumbra, and target mismatch, thereby supporting time-sensitive therapeutic decisions. However, previous comparative studies have shown that different perfusion software packages may provide substantially different core and perfusion lesion volumes, depending on vendor-specific algorithms, deconvolution methods, thresholds, and post-processing pipelines [19,20,21]. This experience illustrates that the clinical adoption of quantitative imaging tools requires not only validation of individual platforms, but also direct assessment of inter-software agreement and interchangeability. A similar issue is now emerging in automated brain volumetry, where multiple commercial tools are increasingly available for clinical use, but their absolute volumetric outputs may not be directly comparable across platforms.

The objective of this study was to assess inter-software variability, agreement, and interchangeability of absolute volumetric outputs from four commercially available AI-based brain volumetry tools applied to identical 3D T1-weighted MRI datasets in patients referred for tremor evaluation. The novelty of this study lies in the direct patient-level comparison of four clinically deployable commercial volumetry platforms on the same clinical MRI examinations, across multiple cortical, subcortical, and CSF-containing regions. While previous studies have commonly focused on the validation of individual commercial tools, comparisons with research-oriented pipelines, or limited pairwise software comparisons, direct head-to-head evaluations among multiple commercially available platforms remain comparatively scarce. Therefore, our study provides practical evidence on the extent to which absolute volumetric outputs from current clinical software packages can or cannot be compared across platforms.

## 2. Materials and Methods

### 2.1. Study Population

This single-center retrospective study included 47 consecutive patients (12 women, 35 men; age range 50–90 years, median age 72.65 +/− 6.53) referred to the Radiology Department of San Salvatore Hospital (L’Aquila, Italy) for MRI evaluation of tremor. Clinical indications included tremor syndromes (38 Parkinson’s Disease, median disease duration 6 (IQR 3–10); 9 Essential Tremor, median disease duration 7 years (IQR 5.5–15)) assessed in routine care, including patients undergoing imaging work-up for possible advanced therapeutic planning [10,11,12]. Inclusion criteria: (i) availability of a brain MRI examination including a high-resolution 3D T1-weighted sequence suitable for volumetric analysis; (ii) adequate image quality on visual quality control. Exclusion criteria: prior neurosurgery; major intra- or extra-axial lesions (e.g., tumor, large territorial infarct, vascular malformation) expected to bias automated segmentation; severe motion or other artefacts precluding reliable processing; or missing 3D T1-weighted data. No specific disease-duration threshold was applied as an inclusion or exclusion criterion for either Parkinson’s disease or essential tremor. Patients were included consecutively if they met the MRI sequence-availability and image-quality criteria; disease duration was recorded descriptively but was not used to restrict cohort selection. The study complied with the Declaration of Helsinki and institutional policies; written informed consent was obtained by all the participants in the study.

### 2.2. Mri Acquisition and Image Processing

All examinations were performed using a 3-tesla MR scanner (MR750w, GE Healthcare, Chicago, IL, USA) with a 32-channel head coil. The protocol included T2 FLAIR (slice 3–0.3, TR 11,000, frequency FoV 24, phase FoV 0.8), T2* GRE (slice 3–0.3, TR 960, frequency FoV 26, phase FoV 0.75), SWI (slice 2 mm, frequency FoV 24, phase FoV 0.85), DWI (slice 3–0.3, TR 10,550, frequency FoV 26, phase FoV 0.8) sequences on axial planes, T2-weighted (slice 3.0–0.3, TR 7854, frequency FoV 26, FoV 26, phase FoV 0.8) sequences on coronal and axial planes, and a volumetric T1-weighted 3D-IR-FSPGR (BRAVO) sequence (slice 1 mm, TR 8.5, frequency FoV 25.6, phase FoV 0.8). A high-resolution 3D T1-weighted acquisition is suitable for quantitative brain morphometry and was used as the input for all volumetric tools [15,16,18]. Images were exported in DICOM format, anonymized, and uploaded to four clinically available, AI-based software packages for automated brain volumetry: mdbrain^®^ version 4.4.1 (mediaire, Berlin, Germany, https://mediaire.ai/en/mdbrain accessed on 28 July 2026), icobrain dm^®^ version 5.13.1 (icometrix, Leuven, Belgium, https://www.icometrix.com accessed on 28 July 2026), Neurophet AQUA^®^ version 3.0.2 (Neurophet Inc., Seoul, Republic of Korea, https://www.neurophet.com/en/solutions/aqua accessed on 28 July 2026), and Pixyl^®^ version 2.1.0 (Pixyl^®^, La Tronche, France, https://pixyl.ai/ accessed on 28 July 2026). The four software packages were selected a priori according to the following criteria: commercial availability and clinical deployability for automated brain MRI volumetry; ability to process routine 3D T1-weighted DICOM data; availability for clinical or research use at our institution during the study period; provision of automated quantitative reports including absolute volumetric outputs for the main regions of interest considered in this study; and minimal or no manual interaction, allowing a reproducible workflow-based comparison. No package was selected according to expected performance. Each software performed fully automated segmentation of cortical and subcortical structures on the 3D T1-weighted data and produced quantitative reports of regional volumes; most packages also provide age-adjusted normative percentiles [15,16,17]. The detailed segmentation algorithms and training datasets used by the commercial packages are proprietary and are not fully disclosed. Nevertheless, the observed differences should be interpreted in the context of known technical variability among automated volumetry tools, including differences in preprocessing, skull stripping, tissue classification, atlas registration, regional parcellation schemes, normative databases, and definitions of lobar or whole-brain boundaries. In particular, lobar volumes may vary according to how each software defines cortical ribbon boundaries, gray–white matter interfaces, sulcal CSF exclusion, and the inclusion or exclusion of adjacent subcortical or infratentorial structures. Recent studies in AI-based medical image analysis have emphasized the importance of standardized preprocessing, reproducible quantitative outputs, and appropriate metric-based evaluation when comparing automated imaging tools. In particular, transformer-based and hybrid deep learning architectures have been increasingly applied to brain MRI classification and segmentation tasks, including limited-data classification frameworks, brain tumor segmentation, and Alzheimer disease prediction models, while quantitative imaging biomarkers such as ADC values have also been evaluated using formal diagnostic performance metrics in PET/MRI settings [22,23,24,25]. These recent methodological developments support the need for transparent reporting of image processing workflows and statistical evaluation metrics when comparing automated volumetric software packages. For the present comparison we extracted the absolute volumes (mL) of prespecified regions: whole brain (parenchymal volume), frontal lobe, parietal lobe, temporal lobe, bilateral hippocampus (summed), and lateral ventricles. When tools provided separate left/right values, hemispheric volumes were summed to obtain bilateral measures. Region coverage differed slightly between platforms; in our dataset Pixyl^®^ did not provide an aggregate occipital lobe volume, so the occipital analysis includes the remaining three tools (see Section 2.4). No manual editing of segmentations was performed. Before software processing, MRI examinations were exported in DICOM format and anonymized. No additional investigator-performed preprocessing, such as manual skull stripping, bias-field correction, registration, intensity normalization, resampling, or manual segmentation correction, was applied. Each software package was used with its default vendor-provided processing pipeline; therefore, any preprocessing required for segmentation was performed internally by the respective proprietary software.

### 2.3. Statistical Analysis

For each region, volumetric measurements from the four software packages were compared using a repeated-measures ANOVA with “software” as the within-subject factor. The sphericity assumption was evaluated using the epsilon estimates provided by MedCalc. When sphericity was not met, Greenhouse–Geisser correction was applied to adjust the degrees of freedom for the omnibus repeated-measures ANOVA. When the omnibus test was significant, pairwise post hoc comparisons were carried out with Bonferroni adjustment for multiple testing, in line with prior comparative studies [15,16].

Agreement across tools was quantified using the intraclass correlation coefficient (ICC) from a two-way mixed-effects model, consistency type, reported as single-measure ICC(3, 1) and average-measure ICC(3, k) with 95% confidence intervals. ICC values were interpreted according to established guidelines: poor <0.50, moderate 0.50–0.75, good 0.75–0.90, and excellent >0.90 [26].To complement ICC analyses, pairwise correlation coefficients were calculated between software outputs for each region to assess preservation of inter-individual ranking. In addition, Bland–Altman analyses were performed to evaluate systematic differences and limits of agreement between software packages. For each pairwise comparison, the mean difference and 95% limits of agreement were calculated as mean difference ± 1.96 standard deviations of the differences [27]. These complementary analyses were included to distinguish correlation or rank-order preservation from absolute measurement interchangeability.

Normality and distributional assumptions were inspected visually; extreme values attributable to processing failure were excluded from sensitivity checks but were not otherwise trimmed. All tests were two-sided with α = 0.05. Analyses were performed with MedCalc statistical software (MedCalc Software Ltd., Ostend, Belgium, version 23.6.1).

### 2.4. Quality Control and Missing Data Handling

A senior neuroradiologist performed post hoc visual quality control for motion/artefacts and segmentation-relevant issues. No external anatomical gold standard was available in this retrospective clinical cohort. Therefore, the present study was designed as an inter-software agreement and interchangeability analysis, rather than as an absolute segmentation accuracy validation study. For this reason, higher or lower absolute volumetric values should not be interpreted as indicating higher or lower accuracy. Because normative percentiles (N%) were not consistently available across platforms and patients, percentile-based summaries were not aggregated; all primary comparisons are based on absolute volumes. For regions not reported by a given tool (e.g., aggregate occipital lobe in Pixyl^®^), we used region-wise complete-case analyses to preserve within-subject across-software comparisons; the number of available cases per region is reported in the Results. Sensitivity checks excluding rare processing failures/outliers yielded the same qualitative conclusions.

### 2.5. Software Outputs and Processing Characteristics

**Figure 1:**
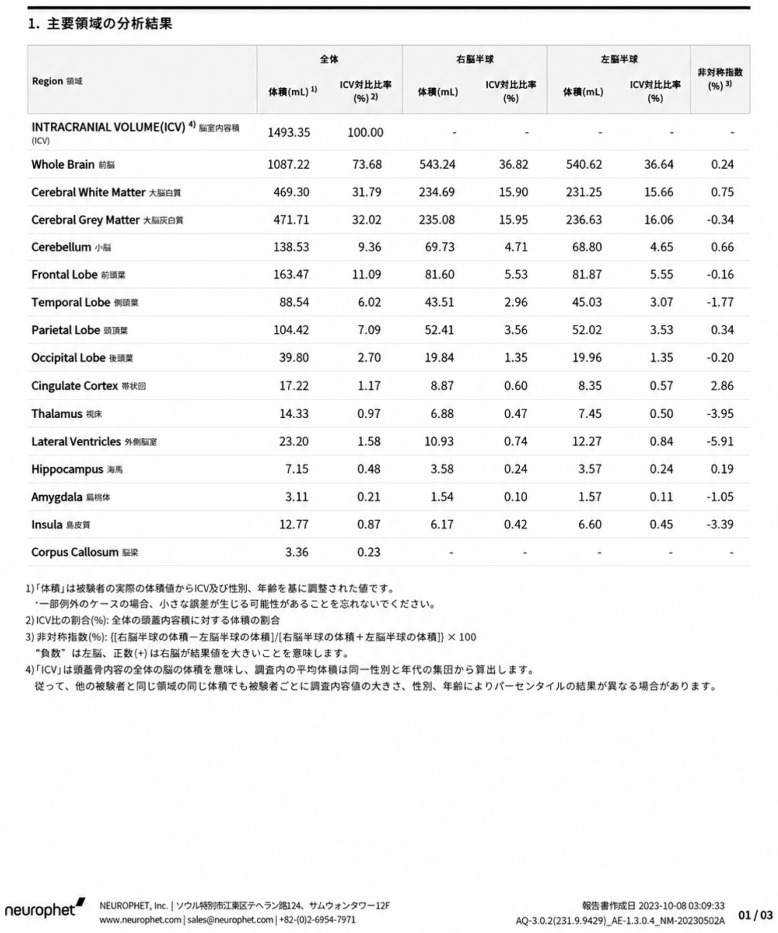
Representative report output of major regional brain volumetric analysis obtained with Neurophet AQUA^®^. The figure is provided for illustrative purposes only, to show the original software-generated report layout and volumetric output. Non-English terms embedded in the original report correspond to standard patient/report labels and do not affect the interpretation of the quantitative information displayed. Any formatting or layout elements embedded in the original vendor-generated report were not used as independent sources of quantitative data for the statistical analyses.

**Figure 2:**
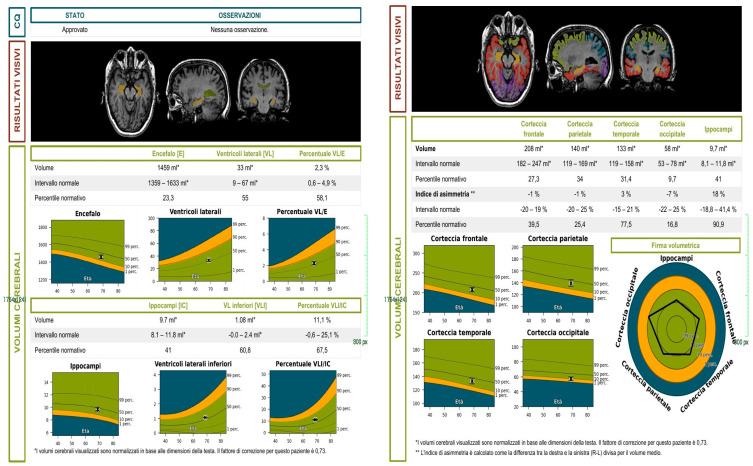
Representative report and segmentation output of different brain areas obtained with icobrain dm^®^. The figure is provided for illustrative purposes only, to show the original software-generated visualization and report layout. The colored overlays correspond to software-generated segmentation maps of different anatomical brain regions; color coding is vendor-defined and illustrative. The report was generated using the Italian-localized version of the software; non-English terms embedded in the original report correspond to standard patient/report labels. Any overlapping or formatting elements embedded in the original vendor-generated report do not affect the interpretation of the segmentation output and were not used for quantitative data extraction or statistical analysis.

**Figure 3:**
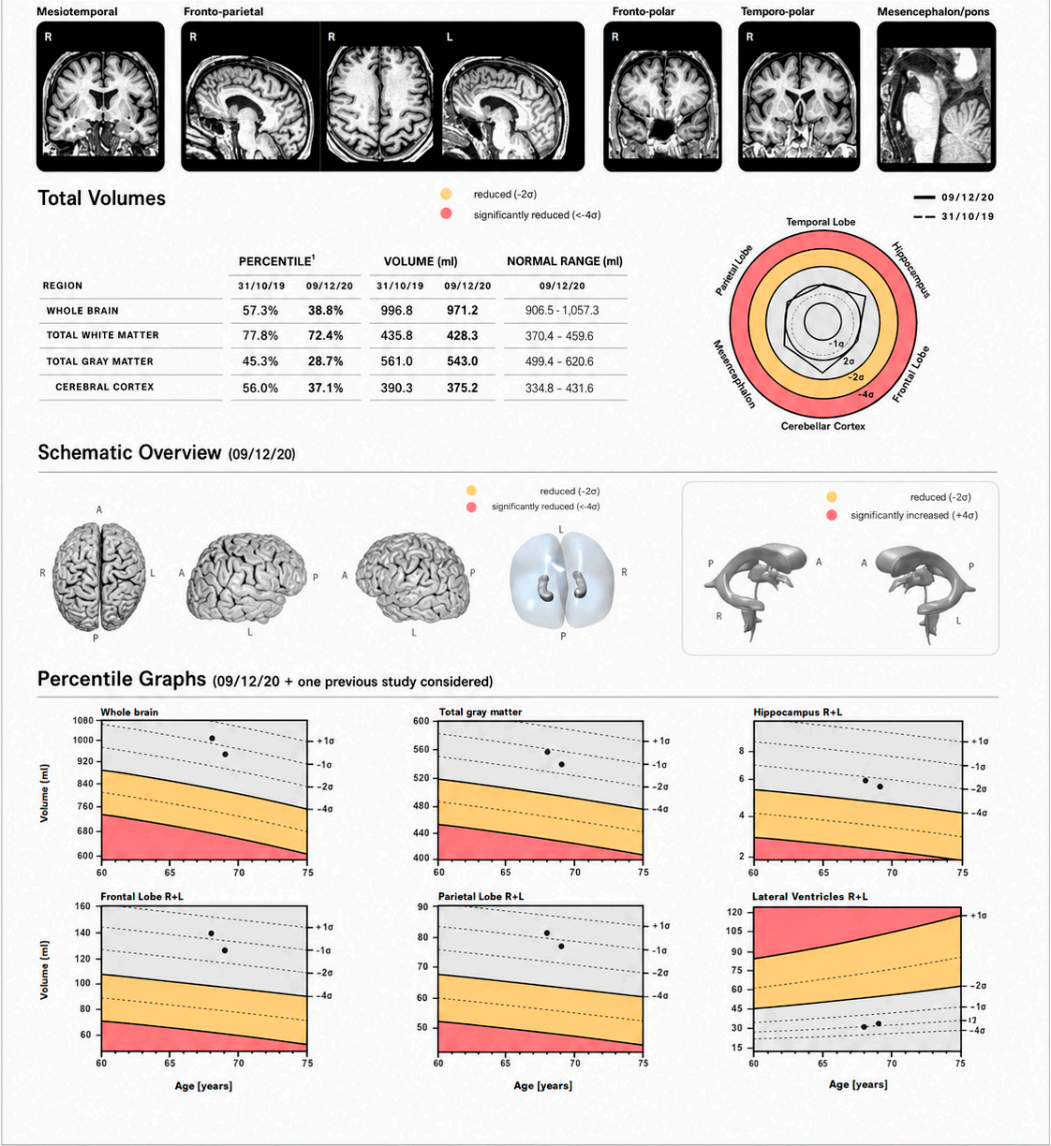
Representative report and segmentation output of different brain areas obtained with mdbrain^®^. The figure is provided for illustrative purposes only, to show the original software-generated visualization and report layout. Colors correspond to vendor-defined segmentation overlays and/or regional volumetric displays generated by the software. Dashed and solid lines represent vendor-generated reference or threshold indicators as displayed in the original report. These graphical elements were not used as independent sources of quantitative data for the statistical analyses.

**Figure 4:**
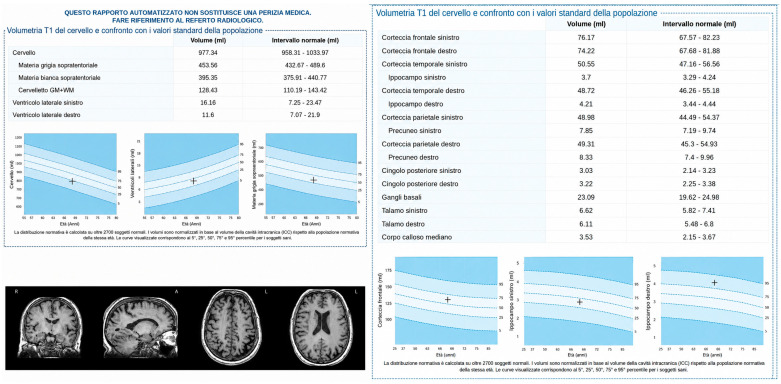
Representative report and segmentation output of different brain areas obtained with Pixyl^®^. The figure is provided for illustrative purposes only, to show the original software-generated visualization and report layout. The left panel shows the first page of the report, including global volumetric measures and representative anatomical images, whereas the right panel shows the second page, including regional volumetric measures and percentile plots. The report was generated using the Italian-localized version of the software; non-English terms embedded in the original report correspond to standard patient/report labels and do not affect the interpretation of the segmentation output. Any formatting or layout elements embedded in the original vendor-generated report were not used for quantitative data extraction or statistical analysis.

Beyond numeric volumes, tools differed in report format, presence of 2D/3D visualizations, and processing times, potentially influencing clinical workflow and user interpretation. These characteristics were not endpoints of the present analysis but are reported qualitatively to contextualize adoption and integration into routine practice [15,16,17]. (see Figure 1, Figure 2, Figure 3 and Figure 4).

## 3. Results

### 3.1. Hippocampus

**Figure 5:**
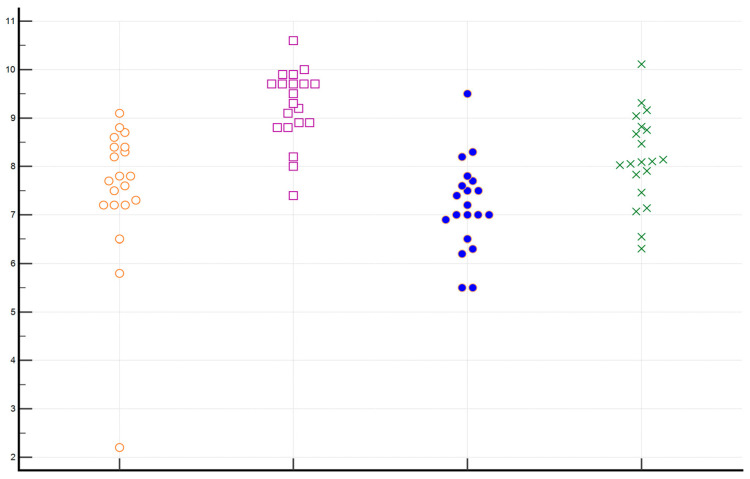
Subject-level hippocampal volumes across software. Swarm/dot plots show individual hippocampal volumes (mL) obtained from four clinically available tools in the same participants (*n* = 47; one dot per subject): Neurophet AQUA (orange circles), icobrain (magenta squares), mdbrain (blue circles), and Pixyl (green crosses). Horizontal jitter is added for readability. A repeated-measures ANOVA indicated a significant main effect of software (*p* < 0.001). Post hoc (Bonferroni-corrected) comparisons showed icobrain > AQUA (*p* = 0.0003), icobrain > mdbrain (*p* < 0.0001), icobrain > Pixyl (*p* < 0.0001); AQUA vs. mdbrain (*p* = 1.000) and AQUA vs. Pixyl (*p* = 0.232) were not significant; mdbrain < Pixyl (*p* < 0.0001). Single-measure ICC = 0.49 (95% CI, 0.27–0.72); average-measure ICC = 0.79 (95% CI, 0.59–0.91).

In the hippocampus, volumetric estimates differed notably among the software (Table 1). icobrain dm^®^ yielded the highest mean hippocampal volume (≈9.3 ± 0.2 mL, 95% CI [8.9–9.6]), substantially larger than the volumes from Neurophet AQUA^®^ (≈7.5 ± 0.3 mL, 95% CI [6.8–8.2]) and mdbrain^®^ (≈7.2 ± 0.2 mL, 95% CI [6.7–7.6]). Pixyl^®^ produced an intermediate hippocampal volume (≈8.2 ± 0.2 mL, 95% CI [7.7–8.6]). Thus, icobrain dm^®^’s hippocampal measurement was about 20–30% higher than the other tools, whereas mdbrain^®^ gave the lowest value. Despite these systematic differences, the inter-software consistency for hippocampal volumes was moderate, with a single-measure intraclass correlation coefficient (ICC) of ~0.49 (95% CI 0.27–0.72) across the four tools. The agreement improved when averaging across methods (ICC ~0.79, 95% CI 0.59–0.91), reflecting good overall reliability. In practice, this indicates that while absolute hippocampal volumes vary by software, the relative ordering of patient volumes is fairly preserved. Icobrain dm^®^ consistently reported higher hippocampal volumes relative to the other tools, whereas Neurophet AQUA^®^ and mdbrain^®^ tended to report smaller hippocampal volumes. (Figure 5; Table 1).

### 3.2. Temporal Lobe

**Figure 6:**
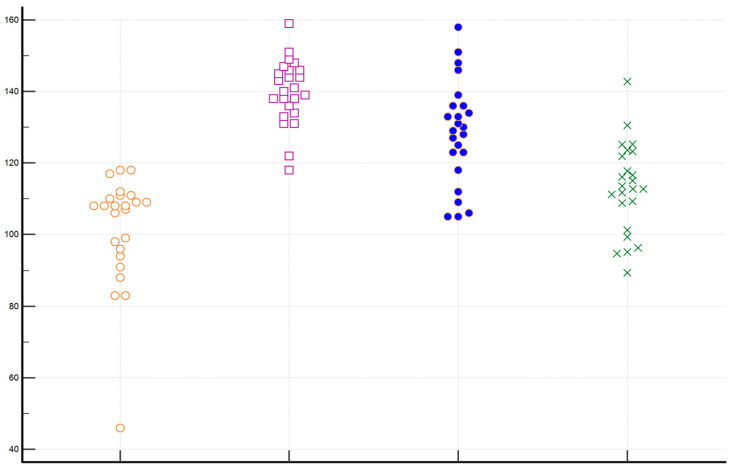
Subject-level temporal lobe volumes across software. Swarm/dot plots show individual temporal lobe volumes (mL) for the same participants (*n* = 47; one symbol per subject): Neurophet AQUA (orange circles), icobrain (magenta squares), mdbrain (blue circles), and Pixyl (green crosses). A repeated-measures ANOVA demonstrated a significant main effect of software (*p* < 0.001). Bonferroni-corrected pairwise tests were significant for the key contrasts: AQUA < icobrain (*p* < 0.0001), AQUA < mdbrain (*p* < 0.0001), AQUA < Pixyl (*p* = 0.0010); icobrain > mdbrain (*p* = 0.0053) and icobrain > Pixyl (*p* < 0.0001); mdbrain > Pixyl (*p* < 0.0001). Agreement was structure-dependent with single-measure ICC = 0.547 (95% CI, 0.347–0.737) and average-measure ICC = 0.828 (95% CI, 0.680–0.918), indicating good-to-excellent concordance in rank ordering but non-interchangeable absolute values.

For the temporal lobe, all four tools reported significantly different volumes. Icobrain dm^®^ again produced the largest mean bilateral temporal lobe volume (≈140.0 ± 1.9 mL, 95% CI [136.2–143.9]), whereas Neurophet AQUA^®^ gave the smallest value (≈101.6 ± 3.2 mL, 95% CI [95.0–108.2]). mdbrain^®^ (≈128.5 ± 3.0 mL, 95% CI [122.4–134.7]) measured a volume slightly lower than icobrain dm^®^’s (by ~9%), and Pixyl^®^ (≈113.1 ± 2.6 mL, 95% CI [107.7–118.4]) reported a volume between the Neurophet AQUA^®^ and mdbrain^®^ estimates. These results indicate that Neurophet AQUA^®^ reported lower temporal lobe volumes (approximately 30% below icobrain dm^®^’s value), whereas icobrain dm^®^ provided considerably higher volumes. The variability in temporal lobe measurements across software was reflected in a single-measure ICC of ~0.55 (95% CI 0.35–0.74), denoting moderate agreement. The ICC for average measurements was ~0.83 (95% CI 0.68–0.92), suggesting that when combining the tools the concordance improves to an excellent range. Accordingly, despite large absolute differences—particularly the lower volumes from Neurophet AQUA^®^—the four methods showed reasonably consistent ranking of temporal lobe volumes across individuals (Figure 6; Table 1).

### 3.3. Parietal Lobe

**Figure 7:**
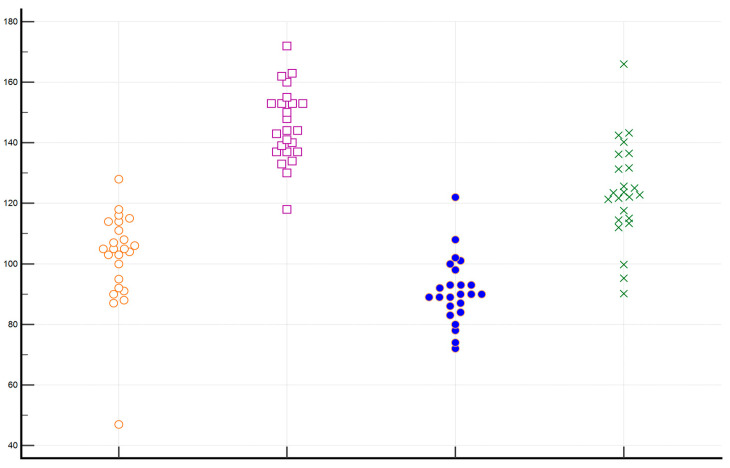
Subject-level parietal lobe volumes across software. Swarm/dot plots display individual parietal lobe volumes (mL) for the same participants (*n* = 47): Neurophet AQUA (orange circles), icobrain (magenta squares), mdbrain (blue circles), and Pixyl (green crosses). A repeated-measures ANOVA showed a significant main effect of software (*p* < 0.001). Bonferroni-corrected pairwise comparisons were all significant except where noted: icobrain > AQUA (*p* < 0.0001), icobrain > mdbrain (*p* < 0.0001), icobrain > Pixyl (*p* < 0.0001); Pixyl > AQUA **(***p* < 0.0001) and Pixyl > mdbrain (*p* < 0.0001); AQUA > mdbrain (*p* = 0.0008). Agreement metrics indicated single-measure ICC = 0.564 (95% CI 0.367–0.749) and average-measure ICC = 0.838 (95% CI 0.698–0.923), consistent with good-to-excellent concordance in ranking despite clear scale offsets.

A similar pattern emerged in the parietal lobe. Icobrain dm^®^ reported a markedly higher mean parietal lobe volume (≈145.8 ± 2.5 mL, 95% CI [140.6–151.0]) compared to the other software. Mdbrain^®^ produced the lowest parietal volume (≈91.0 ± 2.2 mL, 95% CI [86.4–95.6]), about 37% below icobrain dm^®^’s estimate. Neurophet AQUA^®^ measured the parietal lobe at ≈102.2 ± 3.2 mL (95% CI [95.6–108.8]), slightly above mdbrain^®^, while Pixyl^®^ gave an intermediate value (≈123.8 ± 3.4 mL, 95% CI [116.9–130.7]). Thus, for the parietal cortex, mdbrain^®^ and Neurophet AQUA^®^ tended to yield substantially smaller volumes than icobrain dm^®^, with Pixyl^®^ closer to the mid-range. The inter-method agreement for parietal lobe volumes was in the good range (single-measure ICC ~0.56, 95% CI 0.37–0.75; average-measure ICC ~0.84, 95% CI 0.70–0.92). This indicates that while absolute parietal volumes varied (with wider dispersion across tools than some other regions), there was still fairly strong consistency in how each tool ranked patients’ parietal volumes. Notably, icobrain dm^®^ continued to report the highest parietal volumes in this region, whereas mdbrain^®^ reported the lowest parietal volumes relative to the other tools. (Figure 7; Table 1).

### 3.4. Frontal Lobe

**Figure 8:**
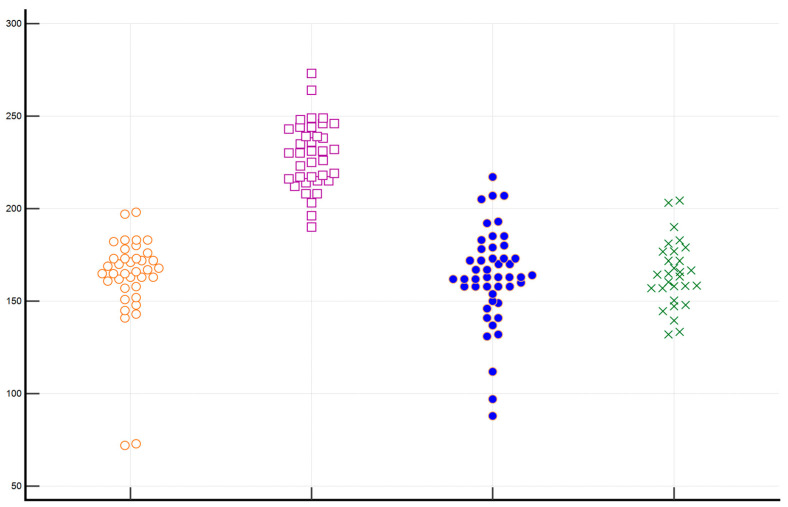
Subject-level frontal lobe volumes across software. Swarm/dot plots show individual frontal lobe volumes (mL) for the same participants (*n* = 47): Neurophet AQUA (orange circles), icobrain (magenta squares), mdbrain (blue circles), and Pixyl (green crosses). A repeated-measures ANOVA demonstrated a significant main effect of software (*p* < 0.001). Bonferroni-corrected pairwise tests: icobrain > AQUA (*p* < 0.0001), icobrain > mdbrain (*p* < 0.0001), icobrain > Pixyl (*p* < 0.0001); contrasts among AQUA vs. mdbrain (*p* = 1.000), AQUA vs. Pixyl (*p* = 1.000), and mdbrain vs. Pixyl (*p* = 1.000) were not significant. Agreement was the lowest among examined regions, with single-measure ICC = 0.431 (95% CI 0.225–0.650) and average-measure ICC = 0.752 (95% CI 0.538–0.881), indicating preserved ranking but non-interchangeable absolute values.

The four software tools showed the greatest divergence in the frontal lobe measurements. Icobrain dm^®^ yielded a mean frontal lobe volume of ≈227.8 ± 4.1 mL (95% CI [219.4–236.2]), substantially higher than the volumes from the other three methods. Neurophet AQUA^®^ and mdbrain^®^ produced nearly identical frontal volumes (≈161–162 mL, with SE ~5 mL; 95% CIs [151.5–170.9] and [151.8–172.6], respectively), which were approximately 30% lower than icobrain dm^®^’s estimate. Pixyl^®^ reported a slightly higher frontal volume than Neurophet AQUA^®^/mdbrain^®^ (≈166.3 ± 3.9 mL, 95% CI [158.3–174.3]), but it remained well below icobrain dm^®^’s value. These findings suggest that icobrain dm^®^ consistently yielded systematically higher volumes for frontal cortex, whereas Neurophet AQUA^®^ and mdbrain^®^ closely agreed and Pixyl^®^ fell in between. The variability and software-dependent offsets in frontal lobe measurements led to the lowest inter-software agreement observed: the single-measure ICC was only ~0.43 (95% CI 0.23–0.65), indicating moderate-to-poor consistency between individual tools for frontal volumes. Even the average-measure ICC reached only ~0.75 (95% CI 0.54–0.88), the lowest among all examined structures (though on the threshold of “excellent” agreement). Thus, the frontal lobe exhibited the highest discrepancy and poorest concordance across software, implying that volumetric outcomes for frontal cortex depend strongly on the software algorithm used. This result is in line with previous reports that some brain regions—particularly large heterogenous lobar structures—show greater between-software measurement variability. (Figure 8; Table 1).

### 3.5. Occipital Lobe

Pixyl^®^ did not give data for the occipital lobe. The absence of Pixyl^®^ outputs for this region highlights a coverage asymmetry between tools that should be taken into account when designing multi-software analyses. Across the three tools that provided occipital measurements, icobrain dm^®^ and mdbrain^®^ yielded comparable volumes (≈66.1 mL vs. ≈64.8 mL; pairwise difference not significant), whereas Neurophet AQUA^®^ reported substantially lower values (≈42.5 mL; *p* < 0.0001 vs. both icobrain dm^®^ and mdbrain^®^). Pixyl^®^ did not provide occipital segmentation in our dataset. This triad pattern—Neurophet AQUA^®^ lower, icobrain dm^®^ and mdbrain^®^ aligned—mirrors the software-specific offsets observed in other lobar regions, while also underscoring that occipital volumes are not directly interchangeable across platforms [15,16,28,29].

### 3.6. Lateral Ventricles

**Figure 9:**
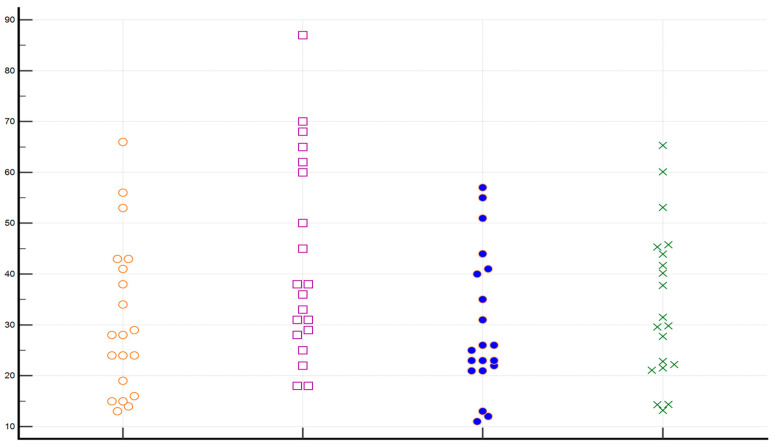
Subject-level lateral ventricle volumes across software. Swarm/dot plots show individual lateral ventricle volumes (mL) for the same participants (*n* = 47): Neurophet AQUA (orange circles), icobrain (magenta squares), mdbrain (blue circles), and Pixyl (green crosses). A repeated-measures ANOVA indicated a significant main effect of software (*p* < 0.001). Bonferroni-corrected pairwise tests showed icobrain > AQUA (*p* < 0.0001), icobrain > mdbrain (*p* < 0.0001), icobrain > Pixyl (*p* = 0.0010); mdbrain < Pixyl (*p* = 0.0029), whereas AQUA vs. mdbrain (*p* = 1.000) and AQUA vs. Pixyl (*p* = 0.314) were not significant. Agreement was excellent, with single-measure ICC = 0.918 (95% CI 0.848–0.963) and average-measure ICC = 0.978 (95% CI 0.957–0.990)**,** indicating robust rank-ordering despite software-dependent scale offsets.

In contrast to the cortical lobes, the lateral ventricles showed the greatest consistency between software tools. Although icobrain dm^®^ measured a somewhat larger ventricular volume (≈42.7 ± 4.4 mL, 95% CI [33.5–51.9]) than the others, the spread was relatively smaller: Pixyl^®^ reported ≈34.1 ± 3.4 mL (95% CI [27.0–41.2]), Neurophet AQUA^®^ ≈31.2 ± 3.4 mL (95% CI [24.0–38.3]), and mdbrain^®^ ≈30.0 ± 3.1 mL (95% CI [23.5–36.5]). Thus, mdbrain^®^ and AQUA closely agreed on ventricular size, Pixyl^®^ was slightly higher, and icobrain dm^®^ provided ~25–40% higher volumes than the other tools. Despite icobrain dm^®^ reporting higher ventricular volumes, all four methods appeared to track ventricular enlargement very coherently across subjects. The lateral ventricles achieved an ICC of ~0.92 for single measures (95% CI 0.85–0.96), the highest of any region, demonstrating excellent agreement. The average-measure ICC was ~0.98 (95% CI 0.96–0.99), indicating near-perfect concordance among the tools for ventricle volumes. In practical terms, this means that even though absolute ventricular volumes differed by software (with icobrain dm^®^ tending higher), each method was highly consistent in identifying patients with larger vs. smaller ventricles. Such strong agreement aligns with expectations, since the lateral ventricles are high-contrast CSF spaces that automated algorithms generally segment reliably. (Figure 9; Table 1).

### 3.7. Total Brain Volume

**Figure 10:**
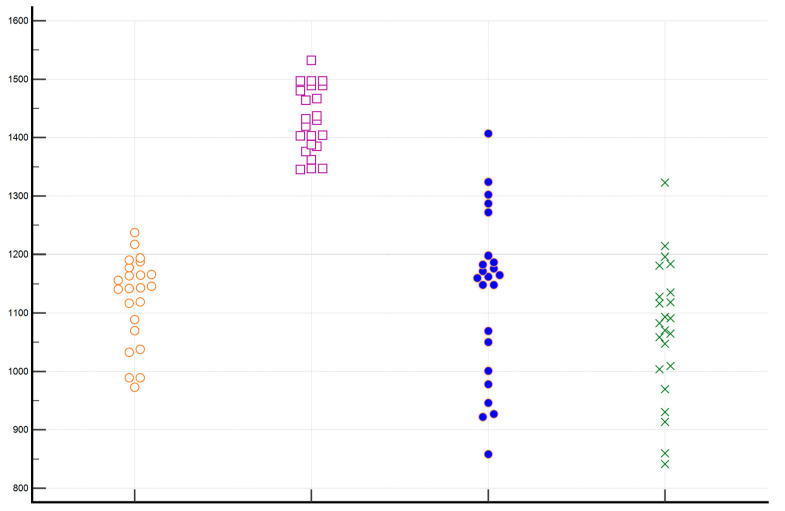
Subject-level total brain volume (TBV) across software. Swarm/dot plots display TBV (mL) for the same participants (*n* = 47; one symbol per subject): Neurophet AQUA (orange circles), icobrain (magenta squares), mdbrain (blue circles), and Pixyl (green crosses). A repeated-measures ANOVA showed a significant main effect of software (*p* < 0.001). Bonferroni-corrected pairwise tests: icobrain > AQUA (*p* < 0.0001), icobrain > mdbrain (*p* < 0.0001), icobrain > Pixyl (*p* < 0.0001); AQUA vs. mdbrain (*p* = 1.000) and AQUA vs. Pixyl (*p* = 1.000) were not significant; mdbrain > Pixyl (*p* = 0.0083). Agreement was moderate at the single-measure level (ICC = 0.513; 95% CI 0.306–0.717) and good for average-measure (ICC = 0.809; 95% CI 0.638–0.910), indicating preserved rank-ordering but non-interchangeable absolute values. Notably, icobrain produced 27–34% larger TBV than the other tools.

Comparisons of total brain volume (TBV)—a global measure encompassing all regions—also revealed systematic software-dependent offsets. Icobrain dm^®^ produced a substantially larger mean TBV (≈1431 ± 11 mL, 95% CI [1407.7–1454.8]) than the other software, consistent with its tendency to report larger absolute volumes. Both Neurophet AQUA^®^ (≈1076.9 ± 49.2 mL, 95% CI [975.2–1178.6]) and Pixyl^®^ (≈1067.2 ± 23.6 mL, 95% CI [1018.4–1115.9]) reported similar total brain volumes—about 25% lower than icobrain dm^®^’s values—whereas mdbrain^®^ gave a slightly higher TBV (≈1129.6 ± 28.7 mL, 95% CI [1070.3–1188.9]) but still well below icobrain dm^®^. This ranking indicates that icobrain dm^®^ included considerably more brain tissue in its segmentation, whereas AQUA and Pixyl^®^ yielded the smallest brain volumes (nearly identical to each other) and mdbrain^®^ was intermediate. The consistency between tools for total brain volume was only moderate. The single-measure ICC was ~0.51 (95% CI 0.31–0.72), reflecting appreciable inter-software variability. The average-measure ICC improved to ~0.81 (95% CI 0.64–0.91), signifying good agreement when considering all methods together. Thus, while all four tools agreed that certain patients had larger brains than others, the magnitude of total brain volume was not directly interchangeable between software packages. These findings echo prior studies that have found significant volume differences in global and regional brain measures between different automated volumetry software, despite often showing high correlations in their measurements. Users should therefore be cautious when comparing absolute brain volumes from different software, as each tool may report systematically higher or lower absolute values depending on its proprietary pipeline and anatomical definitions. (Figure 10; Table 1).

### 3.8. Inter-Software Agreement Summary

Overall, the agreement between the four automated tools ranged from excellent to moderate depending on the structure. The lateral ventricles exhibited the highest concordance (ICC ~0.98 for average measures), followed by the temporal and parietal lobes (average ICC ~0.83–0.84). Total brain volume and the hippocampus showed intermediate agreement (average ICC ~0.79–0.81), while the frontal lobe had the lowest agreement (average ICC ~0.75). In general, larger, well-defined structures (e.g., ventricles) had more consistent measurements across software, whereas complex cortical regions showed greater variability [15,16,17]. Importantly, icobrain dm^®^ showed a distinct measurement profile, reporting the largest absolute volumes across all examined regions relative to the other methods. The remaining three tools (Neurophet AQUA^®^, mdbrain^®^, Pixyl^®^) tended to cluster closer together, though even among them systematic software-dependent offsets were evident (for example, Neurophet AQUA^®^ yielded lower temporal lobe volumes, and mdbrain^®^ gave lower parietal volumes). These systematic offsets underline that volumetric values are not directly interchangeable between different software packages. The particularly large differences observed for frontal lobe and total brain volume may have both anatomical and technical explanations. From a technical perspective, frontal lobe segmentation is challenging because of its complex gyral and sulcal anatomy, broad cortical surface, proximity to the skull base, susceptibility-related signal variations, and partial-volume effects at the gray–white matter and brain–CSF interfaces. Small differences in atlas registration, skull stripping, tissue classification, sulcal CSF exclusion, and lobar boundary definitions may therefore propagate into substantial absolute differences for large lobar regions. Similarly, total brain volume is highly dependent on software-specific definitions of what is included in the global brain mask, such as the inclusion or exclusion of cerebellum, brainstem, deep gray nuclei, periventricular tissue, or borderline CSF/brain voxels. From a biological perspective, age-related atrophy and disease-related regional changes may further increase anatomical heterogeneity in a clinical tremor cohort. Therefore, the larger offsets observed for the frontal lobe and total brain volume likely reflect the combination of complex anatomy, broad region size, and software-specific preprocessing and parcellation strategies. Nonetheless, the high ICCs (generally >0.75) for most regions indicate that all four tools ultimately capture similar inter-individual anatomical patterns across brain structures, despite scale differences. This is consistent with recent comparative studies reporting strong correlations but significant constant offsets between various neuroimaging volumetric software. In summary, each automated tool has its own measurement profile—with icobrain dm^®^ tending to report the largest volumes and others relatively lower—yet their assessments broadly agree on relative differences between brain structures and between patients. This combination of systematic software-dependent offsets and preserved rank-ordering emphasizes the need for calibration when comparing outputs across platforms, while also affirming the reliability of AI-based volumetry in capturing inter-individual brain-structure patterns across software boundaries [28,29]. See Table 1 for a summary of regional volumetry across the different software packages and for regional ICCs and omnibus tests.

Bland–Altman analysis demonstrated substantial inter-software variability in absolute volumetric estimates across brain regions, confirming that measurements obtained from the four automated tools were not directly interchangeable (See Table 2). The largest fixed biases were observed for whole-brain volume, particularly in comparisons involving icobrain, which consistently yielded higher global estimates than AQUA, mdbrain, and Pixyl. A similar pattern was observed for several lobar measures, with icobrain generally providing higher frontal, parietal, and temporal lobe volumes, whereas AQUA tended to yield lower values, especially for occipital and temporal regions. For hippocampal volumes, absolute differences were smaller, but systematic bias remained evident in several comparisons. Lateral ventricular volumes showed comparatively better agreement, with smaller mean differences and fewer proportional biases than cortical and lobar measures. However, proportional bias was frequently detected across anatomical regions, particularly for frontal, temporal, parietal, occipital, and hippocampal volumes, indicating that inter-software disagreement often varied according to the magnitude of the measured volume. Overall, these findings suggest that while software outputs may preserve relative structural trends, absolute volumetric values should not be considered interchangeable across platforms.

### 3.9. Age Correlation Analysis

Correlation analysis with age showed a predominantly inverse relationship between age and brain volumetric measures. Whole-brain volume estimates were significantly negatively correlated with age across all software packages, with the strongest association observed for icobrain (r = −0.719, *p* < 0.0001). Significant negative correlations were also consistently observed for temporal lobe volumes across all tools, as well as for several frontal, parietal, occipital, and hippocampal measurements. Among hippocampal measures, significant inverse correlations were found for mdbrain, Pixyl, and icobrain, whereas AQUA showed only a non-significant trend. Lateral ventricular volumes showed weak positive correlations with age; however, this association reached statistical significance only for Pixyl (r = 0.368, *p* = 0.0496). Overall, these findings indicate that age-related volumetric patterns were generally preserved across software packages, particularly for global and temporal brain volumes, although the strength and significance of correlations varied depending on the anatomical region and software used.

### 3.10. Sex-Based Analysis

Sex-based comparisons showed that male subjects generally had higher volumetric estimates than female subjects across most brain regions and software packages. For whole-brain volume, significantly higher values in males were observed with AQUA, mdbrain, and Pixyl, whereas icobrain did not show a significant sex-related difference. A similar pattern was found for frontal, hippocampal, occipital, parietal, and temporal volumes, with significant male–female differences for most measurements derived from AQUA, mdbrain, and Pixyl. In contrast, icobrain measurements showed no significant sex-related differences across the evaluated regions. Among lobar measures, temporal and frontal volumes showed particularly consistent sex-related differences across software packages, except for icobrain. Parietal volume was significantly higher in males for AQUA and Pixyl, while mdbrain and icobrain did not reach statistical significance. Lateral ventricular volumes did not differ significantly between sexes for any software package. Overall, these findings indicate that sex-related volumetric differences were detected by most software tools for global, lobar, and hippocampal measures, but not for ventricular volumes, with icobrain showing the least sensitivity to sex-related differences in this cohort.

## 4. Discussion

In this head-to-head comparison of four clinically available brain volumetry tools (Neurophet AQUA^®^, icobrain dm^®^, mdbrain^®^, Pixyl^®^), we observed systematic software-dependent offsets in absolute volumes across all examined regions, together with structure-specific variability in agreement. In line with prior multi-tool studies, inter-method rank-ordering of subjects was largely preserved, as reflected by generally good-to-excellent ICCs for most regions, while absolute values remained non-interchangeable [15,16,17,28,29]. Consistency was highest for the lateral ventricles, where high tissue contrast and simpler geometry facilitate robust segmentation across pipelines, and lowest for heterogeneous cortical territories (notably the frontal lobe), where gyral variability and parcellation choices magnify between-tool differences [15,16,28,29]. As previously reported, small subcortical nuclei (e.g., pallidum) can display reduced concordance due to low volumes and boundary ambiguity [16].

Methodologically, several factors plausibly underlie these offsets. Commercial packages differ in pre-processing, registration, atlas definitions, and learning objectives, and they often rely on proprietary normative databases and region lists that are not fully aligned [15,16,17,18,28,29,30]. Consequently, even on identical scans, divergent inclusion/exclusion of cortical ribbon, white-matter border zones, or hippocampal head–tail transitions can propagate into consistent scale shifts. Agreement was quantified using ICCs and complemented by Bland–Altman analyses, as reported in Table 2, to characterize constant and proportional differences between software platforms. These analyses confirmed the presence of systematic and, in several regions, magnitude-dependent inter-software differences, supporting the conclusion that absolute volumetric outputs are not directly interchangeable across platforms. Beyond algorithmic differences, acquisition heterogeneity (field strength, sequence family, head coil) can further widen method gaps; although our cohort was relatively homogeneous, prior work shows that both software choice and scanner/protocol factors contribute to variability in morphometrics [15,16,28,31,32,33]. For longitudinal applications, leveraging unbiased within-subject templates and longitudinal pipelines can reduce measurement noise and measurement variability, but does not eliminate software-specific scaling [34].

The practical implications are immediate. First, diagnostic thresholds and normative percentiles are not portable across software without recalibration; interpretations must remain software-specific [15,16,17,28,29]. Second, for longitudinal monitoring in tremor cohorts, analyses should be platform-consistent over time (and acquisition-stable) to avoid artifactual step-changes that can rival true biological change [15,16,17,28,29,34]. Third, in multicenter research, raw volumetric pooling across platforms risks spurious heterogeneity. Harmonization strategies—statistical (e.g., ComBat-family approaches) and deep-learning contrast/output harmonization—are increasingly effective at mitigating non-biological variance and deserve broader adoption before aggregation or meta-analysis [35,36,37]. Finally, differences we observed in deliverables and coverage (e.g., presence/absence of specific lobar outputs, report layouts, processing times) do not alter the underlying anatomy but do affect workflow, reader confidence, and downstream communication. As a result, a priori standardization of region sets, reporting conventions, and thresholds is advisable when multiple platforms are used within the same program of care or study [15,16,17,28,29,32,33].

The proportional biases identified by the Bland–Altman analyses are particularly relevant for longitudinal monitoring. If the same patient is evaluated over several years using different software platforms, a software-dependent proportional offset could be misinterpreted as accelerated atrophy or apparent stability, especially for large structures such as total brain volume or heterogeneous lobar regions. This effect may be most problematic when baseline and follow-up measurements fall near a clinical or normative threshold, because small biological changes may be amplified or masked by platform-specific scaling. Therefore, extended follow-up should ideally be performed using the same software version and acquisition protocol, and any unavoidable change in platform should be accompanied by local recalibration or harmonization before interpreting individual trajectories [27,34].

These findings have practical implications for both clinical reporting and research design. First, absolute volumetric values obtained from different software packages should not be used interchangeably in individual patients. Second, longitudinal follow-up should ideally be performed using the same software platform and a stable MRI acquisition protocol, in order to avoid artificial changes related to software-dependent measurement offsets. Third, multicenter studies pooling volumetric data from different tools should consider calibration or harmonization strategies before combining absolute values. Finally, in the absence of a gold standard, the choice of software should be guided by the intended clinical use, available regional outputs, workflow integration, regulatory status, transparency of reporting, and local validation, rather than by the assumption that one tool is universally “best”.

These findings may have practical clinical implications in several scenarios. For example, if a patient undergoes baseline volumetry with one software package and follow-up with another, apparent changes in hippocampal, lobar, or total brain volume may reflect software-dependent offsets rather than true biological progression. Similarly, in patients whose regional volumes are close to a normative threshold, different software platforms could potentially classify the same structure differently, influencing the perceived degree of atrophy and the recommended intensity of follow-up. In cognitive or neurodegenerative assessment pathways, non-interchangeable hippocampal or whole-brain volumes may affect radiological interpretation when quantitative reports are used to support visual assessment. In tremor cohorts, volumetry is not usually the sole determinant of treatment eligibility, but discordant estimates of global or regional atrophy may influence the characterization of comorbidity, baseline brain reserve, or suitability for longitudinal monitoring. These examples support platform-consistent follow-up and software-specific interpretation of normative outputs.

Beyond the technical issue of inter-software agreement, standardized AI-derived volumetric outputs may also contribute to future multimodal studies of movement disorders. Tremor syndromes and related movement disorders may include non-motor manifestations, cognitive symptoms, mood disturbances, and systemic comorbidities. In this context, harmonized structural MRI metrics could eventually be integrated with molecular biomarkers, proteomics, metabolomics, and clinical phenotyping to better characterize brain–body interactions and disease mechanisms. Recent multimodal work combining brain imaging and plasma proteomics has shown that imaging–omics fusion can help elucidate systemic pathways linking brain changes, psychiatric symptoms, and metabolic dysfunction [38]. Although such analyses were beyond the scope of the present study, the reliability and harmonization of volumetric MRI outputs are prerequisites for incorporating automated brain volumetry into future multi-omics frameworks in movement disorders.

Beyond proteomic integration, recent multidimensional approaches have also combined morphometric variation with genomic and cell-type-specific expression data to investigate disease comorbidity and neurodevelopmental risk. Such work illustrates how structural brain imaging metrics may serve not only as quantitative anatomical descriptors, but also as intermediate phenotypes linking neuroimaging, molecular biology, and clinical manifestations [39]. In this perspective, standardized and harmonized automated volumetry is a prerequisite for future studies aiming to integrate regional brain structure with multi-omics datasets and to investigate the biological mechanisms underlying motor and non-motor manifestations of movement disorders.

Our study has limitations. There is no in vivo gold standard for regional brain volumes; without expert manual segmentations or physical phantoms we provide a relative inter-method comparison rather than absolute accuracy [15,16,17,28,29]. The use of a single 3-T MRI scanner and a uniform 3D T1-weighted protocol reduced acquisition-related variability and allowed a more direct assessment of software-dependent differences. However, this design also limits generalizability. In multicenter practice, differences in field strength, scanner vendor, head coil, gradient performance, spatial resolution, tissue contrast, signal-to-noise ratio, and reconstruction parameters may interact with preprocessing and segmentation pipelines. Lower spatial resolution or different gray–white matter contrast may increase partial-volume effects and reduce the robustness of tissue classification, whereas scanner-specific intensity profiles may influence bias-field correction, skull stripping, and atlas registration. Therefore, the magnitude of inter-software offsets observed in this single-scanner study may be amplified or modified in heterogeneous multicenter datasets [15,16,28,31]. Coverage asymmetries also represent an important limitation. In particular, the absence of an aggregate occipital lobe output from Pixyl^®^ prevented a full four-way comparison for this region and limits the generalizability of the occipital findings relative to the other lobar regions. Importantly, this limitation does not affect the four-software comparisons performed for hippocampus, temporal, parietal and frontal lobes, lateral ventricles, and total brain volume, but it illustrates a broader practical issue: commercial volumetry platforms may differ not only in their absolute measurements but also in the anatomical regions made available to the user. Such coverage disparities should be considered when selecting software for clinical follow-up or multicenter research, because missing or non-harmonized regional outputs may prevent direct pooling across platforms [15,16,17,28,29]. Head-coil configuration and gradient performance may also interact with proprietary preprocessing steps in ways that are difficult to predict. Different head coils can alter receive-field profiles, signal-to-noise ratio, and intensity non-uniformity, potentially affecting bias-field correction, skull stripping, and tissue classification. Similarly, differences in gradient performance may influence geometric fidelity, distortion, and effective spatial resolution, thereby modifying partial-volume patterns at gray–white matter and brain–CSF interfaces. Because each commercial package implements its own quality-control, normalization, and segmentation pipeline, these hardware-related effects may be compensated for differently across tools. This hardware–software interaction should therefore be considered when pooling multicenter data acquired with different scanners, coils, and acquisition protocols [15,16,28,31].

Despite these constraints, our findings are concordant with recent comparative literature and likely reflect class-level behavior of current clinical volumetry tools.

## 5. Conclusions

In conclusion, four commercially available AI-based brain volumetry tools applied to identical 3D T1-weighted MRI examinations produced significantly different absolute volumetric outputs across multiple brain regions. Agreement was structure-dependent, being highest for lateral ventricles and lower for complex lobar regions [15,16,28,29]. Because no external anatomical gold standard or expert manual segmentation masks were used, our results do not establish which software is most accurate. Rather, they demonstrate that absolute volumetric values are not directly interchangeable across platforms, although relative subject ranking is partly preserved [15,16,17,28,29,32,33]. Therefore, our findings should be interpreted as evidence of partial inter-method consistency in subject ranking, not as proof of absolute reliability or segmentation accuracy of any individual platform. Platform consistency is therefore recommended for longitudinal monitoring, and calibration or harmonization strategies should be considered in multicenter or multi-software studies [15,16,17,28,29,34,35,36,37]. Future validation studies should include expert-derived reference masks and voxel-wise accuracy metrics to determine the absolute segmentation performance of each platform [32,33].

## Figures and Tables

**Table 1:** Summary of regional volumetry across software. Values are mean ± SE [95% CI]. n denotes complete-case sample size per region. ICC: two-way mixed, consistency (single/average). Omnibus *p*: RM-ANOVA (Greenhouse–Geisser corrected where applicable). Occipital: three-tool comparison; ICC not reported due to heterogeneous coverage.

Region	Neurophet AQUA^®^	Icobrain Dm^®^	Mdbrain^®^	Pixyl^®^	ICC Single (95% CI)	ICC Average (95% CI)	Omnibus RM-ANOVA (*p*)
Hippocampus	7.52 ± 0.33 [6.82–8.21]	9.25 ± 0.17 [8.89–9.61]	7.18 ± 0.21 [6.74–7.62]	8.15 ± 0.21 [7.71–8.59]	0.49 (0.27–0.72)	0.79 (0.59–0.91)	<0.001
Temporal	101.58 ± 3.20 [95.0–108.2]	140.04 ± 1.85 [136.2–143.9]	128.54 ± 2.95 [122.4–134.7]	113.08 ± 2.59 [107.7–118.4]	0.55 (0.35–0.74)	0.83 (0.68–0.92)	<0.001
Parietal	102.17 ± 3.20 [95.55–108.78]	145.79 ± 2.49 [140.63–150.95]	90.96 ± 2.21 [86.36–95.55]	123.80 ± 3.35 [116.86–130.73]	0.56 (0.37–0.75)	0.84 (0.70–0.92)	<0.001
Frontal	161.21 ± 4.70 [151.49–170.93]	227.83 ± 4.06 [219.44–236.23]	162.21 ± 5.05 [151.77–172.65]	166.29 ± 3.85 [158.32–174.26]	0.43 (0.23–0.65)	0.75 (0.54–0.88)	<0.001
Occipital †	42.5 (approx)	66.1 (approx)	64.8 (approx)	—	—	—	<0.001
Lateral ventricles	31.15 ± 3.42 [23.99–38.31]	42.70 ± 4.41 [33.46–51.94]	30.00 ± 3.10 [23.52–36.48]	34.06 ± 3.40 [26.96–41.17]	0.92 (0.85–0.96)	0.98 (0.96–0.99)	<0.001
Total brain volume	1076.88 ± 49.17 [975.16–1178.59]	1431.25 ± 11.36 [1407.74–1454.76]	1129.58 ± 28.65 [1070.31–1188.85]	1067.15 ± 23.58 [1018.38–1115.92]	0.51 (0.31–0.72)	0.81 (0.64–0.91)	<0.001

**Table 2:** Bland–Altman analysis of inter-software agreement across brain regions. Mean bias, 95% limits of agreement, and proportional bias are reported for each pairwise comparison between automated volumetric software packages. Bias is expressed as Method A − Method B. LoA = limits of agreement.

Region	Comparison	Mean Bias	95% LoA	Proportional Bias
Whole brain	AQUA − icobrain	−330.9	−608.4 to −53.4	*p* < 0.0001
Whole brain	AQUA − mdbrain	−43.4	−318.8 to 231.9	*p* = 0.0001
Whole brain	AQUA − Pixyl	34.9	−176.6 to 246.5	*p* = 0.0069
Whole brain	icobrain − mdbrain	283.9	24.5 to 543.3	*p* = 0.272
Whole brain	icobrain − Pixyl	364.1	151.9 to 576.3	*p* = 0.567
Whole brain	mdbrain − Pixyl	66.2	−85.6 to 218.1	*p* = 0.0022
Frontal lobe	AQUA − icobrain	−66.3	−114.1 to −18.5	*p* < 0.0001
Frontal lobe	AQUA − mdbrain	1.3	−54.2 to 56.9	*p* = 0.0009
Frontal lobe	AQUA − Pixyl	−5.1	−41.9 to 31.8	*p* = 0.0016
Frontal lobe	icobrain − mdbrain	67.0	12.5 to 121.6	*p* = 0.0164
Frontal lobe	icobrain − Pixyl	61.5	15.4 to 107.6	*p* = 0.0009
Frontal lobe	mdbrain − Pixyl	−1.1	−45.6 to 43.4	*p* < 0.0001
Hippocampus	AQUA − icobrain	−1.77	−4.98 to 1.43	*p* < 0.0001
Hippocampus	AQUA − mdbrain	0.25	−2.55 to 3.05	*p* < 0.0001
Hippocampus	AQUA − Pixyl	−0.60	−2.93 to 1.74	*p* < 0.0001
Hippocampus	icobrain − mdbrain	2.10	0.56 to 3.64	*p* = 0.103
Hippocampus	icobrain − Pixyl	1.00	−1.06 to 3.07	*p* = 0.0023
Hippocampus	mdbrain − Pixyl	−0.97	−1.42 to −0.52	*p* = 0.667
Occipital lobe	AQUA − icobrain	−22.65	−44.49 to −0.81	*p* < 0.0001
Occipital lobe	AQUA − mdbrain	−22.61	−51.45 to 6.24	*p* = 0.0001
Occipital lobe	icobrain − mdbrain	−0.11	−28.59 to 28.37	*p* = 0.0066
Parietal lobe	AQUA − icobrain	−43.97	−78.38 to −9.57	*p* < 0.0001
Parietal lobe	AQUA − mdbrain	12.50	−20.09 to 45.09	*p* < 0.0001
Parietal lobe	AQUA − Pixyl	−21.63	−49.35 to 6.09	*p* = 0.0551
Parietal lobe	icobrain − mdbrain	56.19	27.77 to 84.61	*p* = 0.0002
Parietal lobe	icobrain − Pixyl	22.00	−8.85 to 52.84	*p* = 0.1139
Parietal lobe	mdbrain − Pixyl	−32.62	−45.19 to −20.05	*p* = 0.0001
Temporal lobe	AQUA − icobrain	−38.78	−68.63 to −8.93	*p* < 0.0001
Temporal lobe	AQUA − mdbrain	−26.11	−57.97 to 5.76	*p* = 0.0076
Temporal lobe	AQUA − Pixyl	−11.50	−36.11 to 13.11	*p* = 0.0013
Temporal lobe	icobrain − mdbrain	12.22	−21.56 to 45.99	*p* = 0.0966
Temporal lobe	icobrain − Pixyl	26.96	0.92 to 53.00	*p* = 0.0475
Temporal lobe	mdbrain − Pixyl	15.35	8.14 to 22.56	*p* = 0.0006
Lateral ventricles	AQUA − icobrain	−12.14	−31.94 to 7.67	*p* = 0.9442
Lateral ventricles	AQUA − mdbrain	0.30	−13.59 to 14.19	*p* = 0.0057
Lateral ventricles	AQUA − Pixyl	−2.56	−13.96 to 8.83	*p* = 0.2563
Lateral ventricles	icobrain − mdbrain	12.91	−0.47 to 26.29	*p* < 0.0001
Lateral ventricles	icobrain − Pixyl	8.69	−7.07 to 24.45	*p* = 0.0005
Lateral ventricles	mdbrain − Pixyl	−4.06	−12.55 to 4.43	*p* = 0.4807

## Data Availability

The data that support the findings of this study are available from the corresponding author, upon reasonable request.
